# Emotional Intelligence and Its Relationship with Subjective Well-Being and Academic Achievement in University Students

**DOI:** 10.3390/jintelligence13040042

**Published:** 2025-03-26

**Authors:** Presentación Ángeles Caballero-García, Sara Sánchez Ruiz

**Affiliations:** Faculty of Education, Camilo José Cela University, 28692 Madrid, Spain; ssanchez@ucjc.edu

**Keywords:** emotional intelligence, subjective well-being, academic achievement, soft skills, higher education, employability

## Abstract

The demands of the labor market are a constant challenge for universities, emphasizing the crucial importance of competency-based education to make our students more academically and professionally competitive. The benefits of Emotional Intelligence (EI) and Subjective Well-Being (happiness/life satisfaction) (SWB) have been evidenced as necessary competencies in personal, academic, and professional contexts. Our research assessed these variables in a sample of 300 university students from Madrid (Spain), comprising 68 (22.7%) men and 232 (77.3%) women, aged between 18 and 47 years (M = 21.72; SD = 0.42). Our objective was to determine their baseline levels, study their relationship with Academic Achievement (AA), analyze their changes after a positive emotional intervention, and determine if they are predictors of AA. For this, we used a quasi-experimental pre/post-test design with experimental/control groups. Our results show medium–high baseline levels of EI, SWB, and AA in our students; positive correlations, which improved in intensity in the post-test, between EI (clarity and repair) and SWB, between AA and EI (attention), and between AA and happiness (OHI); and better scores in EI and happiness in the post-test compared to the pre-test, and in the experimental group compared to the control group, as a result of our intervention. Finally, the findings indicate that EI (attention) and SWB (life satisfaction) jointly predict a small part of AA. The data are discussed for their implications for change in higher education, towards competency-based education interventions that improve the outcomes and employability of our students and bridge the university/industry gap.

## 1. Introduction

To ensure the quality, comparability, and effectiveness of higher education in Europe, in 1999, the Tuning Project provided universities in the European Higher Education Area with methodologies to align their educational programs with academic and professional standards (González and Wagenaar 2005).

Subsequently, the European Qualifications Framework (EQF) translated these principles for higher education and, in 2008, unified the national university qualifications across Europe, emphasizing the development of skills and professional competencies over mere knowledge (Méhaut and Winch 2012).

However, the latest report (European Education and Culture Executive Agency 2024) indicates that, although significant progress has been made, there are still important challenges in terms of competency-based education and its alignment with labor market needs. This is crucial for progress and poses a significant challenge for universities, considering that higher education is the stage that precedes the world of work.

Like universities, companies also seek candidates who, in addition to academic and technical knowledge, possess soft skills such as effective communication, teamwork, problem-solving, adaptability, leadership, critical thinking, Emotional Intelligence (EI), and the management of Subjective Well-Being (SWB), among others. These skills are crucial for success in any career and are highly valued in today’s society and labor market (Chen et al. 2024; Garavito-Hernández et al. 2024; Mwita et al. 2023; Ragusa et al. 2022; Velásquez et al. 2024).

However, their limited presence in university curricula is surprising (Cizelj 2024; Saravia Domínguez et al. 2024; Wagenaar 2019). This need has led us to evaluate certain cognitive and emotional management skills (Garavito-Hernández et al. 2024) in our environment, such as EI and SWB (in their components of happiness and life satisfaction), in an attempt to understand the competency levels of our students in these variables and demonstrate that their development is both possible and desirable, benefiting educational quality and reducing the previously mentioned university/business gap.

The development of EI at university is fundamental because it not only improves the social and mental well-being of students (García Morales 2022) but also provides them with the necessary skills to face conflict situations and make sound decisions in their daily lives (Puertas-Molero et al. 2020).

Systematic study mappings such as those by Espinoza Mina and Gallegos Barzola (2020) and recent meta-analyses like those by MacCann et al. (2020), Sánchez-Álvarez et al. (2020a, 2020b), among others, also highlight the crucial role of EI for academic and professional success, considering that it facilitates adaptation to the environment and the management of one’s own and others’ emotions.

For all these reasons, integrating EI into university programs is a significant challenge for us, considering that it not only prepares students for the labor market but also contributes to their personal and social development, promoting a healthier and more productive educational environment while making them more competitive in their access to the labor market.

Since Mayer and Salovey (1990) introduced the term EI in the scientific field, several explanatory models have been developed (Mancini et al. 2022). Ability models, based on Salovey and Mayer’s conception, understand IE as the ability to perceive, understand, and regulate emotions in oneself and others, and assess it as a cognitive ability using performance instruments. Trait models, such as those described by Petrides et al. (2007), conceive EI as a personality trait and constellation of emotional perceptions assessed with rating instruments. Finally, mixed models, such as those of Goleman (1995) and Bar-On (1997), consider EI as a broader set of skills, mixing traits and aptitudes in their measurement.

In our research, we chose to study EI using Mayer and Salovey’s (1990) model, evaluating the metaknowledge of three emotional states: attention (the degree to which individuals perceive and focus on their own emotions and feelings), clarity (the ability to understand and clearly distinguish one’s own emotions), and emotional repair (the ability to effectively manage and control one’s own emotions). This model is one of the most accepted due to its coherence, feasibility, and theoretical and scientific rigor and because, as Fernández-Berrocal and Extremera-Pacheco (2005) point out, it facilitates the development of viable and evaluable intervention programs, which was our intention.

Regarding the empirical science of SWB, popularly known as happiness or life satisfaction, we want to highlight that it has grown enormously in the last decade and plays a significant role in universities due to its link with mental health and academic success (Diener et al. 2018), like EI.

The concept of SWB was introduced by Wamer in 1967. There are as many definitions as there are studies on the subject (Muñoz Umaña 2007). Consequently, different explanatory models have emerged, traditionally classified into two main approaches (Vielma Rangel and Alonso 2010): the hedonic approach, focused on life satisfaction and the experience of positive/negative emotions, with Diener (subjective well-being), Csikszentmihalyi (flow), and Kahneman (experienced and remembered well-being) as some of its main representatives, and the eudaimonic approach, centered on the development of human potential and personal fulfillment, with Ryff (psychological well-being), Deci and Ryan (self-determination), Seligman (PERMA), Huppert and others (flourishing) as notable authors. Additionally, integrative approaches have emerged, combining elements of both to offer a more comprehensive and multidimensional view (Cobo-Rendón et al. 2020), supported by authors such as Corey Keyes (social well-being) and Jahoda (positive mental health).

These approaches and models provide a solid basis for research and practice in the field of SWB, allowing for a broader and more nuanced understanding of the concept. However, the most widely accepted classification in the scientific literature in the fields of psychology and social sciences is still the division into two main components that Diener gave in 1984: cognitive, referring to life satisfaction, as an overall assessment of a person’s quality of life according to their own criteria, and affective, linked to happiness, which includes the presence of positive affects and the absence of negative affects (Muñoz Umaña 2007). In their systematized review of the literature, Diener et al. (2018) confirms that this is the most comprehensive, robust, and widely used view, which is why it has served as the basis for our research.

Diving deeper into the literature on baseline levels of our variables in the context of higher education, where our study takes place, we have observed that university students tend to have medium–high or moderate levels of EI (Rábago de Ávila et al. 2019; Gonzaga et al. 2024; Llanos Bardales et al. 2023), happiness (Liang and Sun 2022; Rábago de Ávila et al. 2019; Ross et al. 2019; Savari et al. 2023), life satisfaction (Abdullah et al. 2022), and Academic Achievement (AA) (Barrera Hernández et al. 2018; Niño-González et al. 2017; Sánchez-Ruiz 2020).

However, despite the average results being good, there are deficiencies in these competencies that students need to improve, considering that they help, as we have noted, to manage stress and academic pressures, achieve emotional balance in negative situations (Hollerer and Kohl 2022), and face academic, personal, and professional challenges more effectively, thereby promoting their overall success and well-being (Brackett et al. 2011; MacCann et al. 2020). This justifies their assessment and intervention for development in the classroom and the examination of their effects on AA, as proposed in this research.

EI, happiness, life satisfaction, and AA are interrelated variables (Puertas-Molero et al. 2020). Empirical evidence has shown that the relationship between EI and SWB tends to be positive (Ye et al. 2024), and ranges from low (Akdeniz and Yaizi 2023) to moderate (Llamas-Díaz et al. 2023; Sánchez-Álvarez et al. 2020a, 2020b; Trang et al. 2023), especially with the dimensions of emotional clarity and repair (Hidalgo-Fuentes et al. 2022), and negative and weak in the case of emotional attention. This indicates that students with good understanding and management of emotions also perceive themselves with greater well-being, increasing their chances of success in different areas of life. SWB is not strongly favored when exaggerated attention is paid to emotions (Blasco-Belled et al. 2019) but is positively influenced by emotional repair (Cañero Pérez et al. 2019; García Morales 2022; Tejada-Gallardo et al. 2022).

Higher levels of happiness correlate positively with higher life satisfaction, and the relationship is often moderate (Çakir and Demirel 2019). Students with higher EI are also more optimistic about life events and problems, better able to face daily challenges, and more satisfied with their lives. This creates a positive work environment and improves their academic success (Diener et al. 2018; Fernández-Berrocal et al. 2004). Therefore, it is of great interest to incorporate these cognitive–emotional competences into university curricula, considering that they will influence both the psychological well-being and the academic, social, and professional performance of students (Alonso-Aldana et al. 2020).

This has led to an increase in research on SWB and other cognitive–emotional variables such as EI in university students in recent years. In their systematized review of the literature, Cobo-Rendón et al. (2020) noted that, despite this, much remains to be developed. Research has focused more on testing its relationship with positive variables, social impact, and mental health, and less on academic variables, which is why we have included AA in our analysis.

Reviewing the literature on the relationship between EI and SWB with AA in university students, we have found that the data partially support this relationship and offer uneven results.

Most studies that have analyzed the relationship between EI and AA find positive correlations between these variables (Altwijri et al. 2021; Khan et al. 2023; Llanos Bardales and Machuca Cabrera 2023; Racu and Dranga 2023; Rauf and Iqbal 2024; Sánchez-Álvarez et al. 2020a; Sánchez-Bolívar et al. 2023; Ye et al. 2024; Tingyu et al. 2024; Ubago-Jimenez et al. 2024) and have demonstrated the predictive nature of EI, which explains between 1.7 and 2.3% of the variance in AA (MacCann et al. 2020). A recent meta-analysis (Llanos Bardales and Machuca Cabrera 2023) concluded that 71.4% of the reviewed studies confirm this positive and moderate correlation.

However, others have not been able to demonstrate this relationship (Arntz Vera and Trunce Morales 2019; Bilimale et al. 2024; Juyal et al. 2023), suggesting that the relationship may be more complex and not always significant, opening new avenues for research (Sánchez-Álvarez et al. 2020b).

Similarly, with happiness, some authors show a positive and significant relationship between it and AA (Alves et al. 2019; Harrison et al. 2024; Jiang et al. 2022; Khan et al. 2020; Khodabakhsh et al. 2019; Moussa and Ali 2021; Ravina-Ripoll et al. 2019). Conversely, others have not been able to demonstrate this relationship (Savari et al. 2023). Ramírez and Fuentes (2013) confirmed the positive and significant effect and the predictive nature of happiness on AA, although this prediction represents only a small part of the total variability of AA, considering that the effect is moderate and represents only 2.5% of the total variability. In Salehi et al. (2013) work, happiness explained only 13% of the variance in AA. This small but interesting explanation underscores the importance of happiness in the academic context, although further research is needed to better understand its predictive effects on AA (Osornio Castillo et al. 2011).

The relationship between life satisfaction and AA in university students has also been reported by several authors (Perveen et al. 2021; Salanova et al. 2005; Ye et al. 2024), although a minority of authors have failed to find statistical significance (Cordero 2024). Research such as that of Akin and Akin (2016) confirms that life satisfaction is positively related to and a predictor of AA. Although it explains only a small part of the total variability in AA (3%) and the effect size is small, it is statistically significant.

The contradictory results found for each of the variables we have evaluated invite further research in this area. The complexity of these interactions and the moderate effects observed indicate that there is much more to discover and understand, which can have important implications for the development of educational interventions and student support programs in higher education (Llanos Bardales and Machuca Cabrera 2023) in which we have been interested.

EI and SWB are skills that can be learned and developed through specific interventions (Brackett et al. 2011; Durlak et al. 2011; Fernández-Berrocal et al. 2022; Quílez-Robres et al. 2023; Puertas-Molero et al. 2020). In our review of the literature on intervention programs in the university context, we have found that they are effective and improve EI (Quílez-Robres et al. 2023; Taylor et al. 2022), SWB (Ye et al. 2024; Suárez and Marrero 2020), and AA (Bolier et al. 2013; Caballero-García and Sánchez-Ruiz 2018, 2021, 2024; Caballero-García et al. 2019; Sánchez-Ruiz 2020; Puertas-Molero et al. 2020).

Working on these ’soft skills’ is fundamental in higher education; however, their implementation is still limited (Durlak 2016; Durlak et al. 2011; Extremera-Pacheco and Rey 2016; Sánchez-Ruiz 2020; Stallman and Kavanagh 2018) compared to other stages or groups (Brackett et al. 2011; Fernández-Berrocal et al. 2022), hence their necessity.

The purpose of our research was precisely to understand the levels of EI, SWB (happiness/life satisfaction), and AA among our students; to find out if these variables were related to each other, especially after an intervention for their development; to examine the effects of this intervention on these variables in the experimental group compared to the control group; and to study the predictive nature of EI and SWB on AA, in order to promote learning environments at the university that work on these competencies as facilitators of performance and employability, as well as personal and social development.

In this context, our research hypotheses were expected to find average levels of EI, SWB, and AA; positive relationships between these variables, especially after the intervention; positive and significant effects of this intervention on EI, SWB, and AA; and the predictive nature of EI and SWB on AA.

## 2. Methodology

To achieve these research objectives and hypotheses, we employed a quantitative, cross-sectional methodology with a quasi-experimental design, using a non-equivalent control group and pre-test/post-test measures (Campbell and Stanley 1966; Cook and Campbell 1979; León and García-Celay 2015).

### 2.1. Participants

The sample comprised 300 university students, 68 (22.7%) men and 232 (77.3%) women, aged between 18 and 47 years (M = 21.72; SD = 0.419). They were selected non-probabilistically and intentionally, according to the availability of participants and ease of access, from a population of 230,563 enrolled in public and private universities in the Community of Madrid (Spain). To ensure the representativeness of the sample and the accuracy of our estimates, we calculated the minimum necessary sample size (254 students) and the maximum sampling error assumed with this selection (López-Roldán and Fachelli 2015). The final sample exceeded the estimated size by 8.11% and proved to be adequate (Kleeberg-Hidalgo and Ramos-Ramírez 2009), with an acceptable margin of error (5.65%) and a confidence level of 95%. The experimental group consisted of 162 students (54%), 122 women (40.7%) and 40 men (13.3%), and the control group consisted of 138 students (66%), 110 women (36.7%) and 28 men (9.3%). The study also included 6 female university professors, aged between 30 and 60 years, with extensive teaching and research experience, who received specific training in positive emotional education, worked with the experimental groups, designed activities for positive emotional education, and integrated them into their subjects for the duration of the intervention.

### 2.2. Instruments

The Trait Meta-Mood Scale (TMMS-24) is a shortened version of the TMMS-48 (Salovey et al. 1995), adapted to Spanish by Fernández-Berrocal et al. (2004). It evaluates 3 dimensions of perceived EI: attention, clarity, and emotional repair, with 8 items each and a 5-point Likert scale (from 1—strongly disagree to 5—strongly agree), with adequate psychometric properties—attention (α = 0.90), clarity (α = 0.90), and repair (α = 0.86) (Extremera-Pacheco et al. 2004). In our study, the values ranged between 0.79 and 0.82 (attention: α = 0.82, ω = 0.82; clarity, α = 0.79, ω = 0.78; repair, α = 0.80, ω = 0.79; and total EI, α = 0.78, ω = 0.79), indicating good internal consistency (Kaplan and Saccuzo 2009; Murphy and Davidshofer 2005; Celina Oviedo and Campo-Arias 2005). It was selected for its suitability to the reference model, widespread use, simplicity (Fernández-Berrocal et al. 2023; Javaid et al. 2024; Suyo-Vega et al. 2023), and empirical evidence of reliability and validity (Arrivillaga and Extremera 2020), both in previous studies and in our research.

The Subjective Happiness Scale (SHS) was created by Lyubomirsky and Lepper (1999). It evaluates happiness as an emotional component of SWB and a molar category of emotional psychological well-being, beyond the sum of positive/negative emotional states and cognitions related to the phenomenon, from the respondent’s perspective, using 4 Likert-type items on a 7-point scale (from 1—not at all to 7—to a great extent). The original scale was validated in American and Russian samples (Lyubomirsky and Lepper 1999). It has Chinese, Malay, German, and Filipino versions, which replicate the unidimensional structure of happiness and show good internal consistency and convergent validity with other SWB measures (Swami et al. 2009). It has also been widely used with Spanish populations (Extremera-Pacheco et al. 2011), with a reliability of 0.72–0.80, and Spanish-speaking populations (Gutiérrez-Cobo et al. 2016), with a reliability of 0.78–0.94. Therefore, it meets the psychometric criteria of reliability and internal consistency (between 0.79 and 0.94), test–retest reliability (between 3 weeks and 1 year, varying between 0.55 and 0.90), convergent validity (between 0.52 and 0.72 with other happiness measures) and divergent validity with the Beck Depression Inventory, Big-Five questionnaire, and dispositional optimism (LOT-R), as well as highly significant levels of factorial reliability (between 0.73 and 0.87) and temporal stability (0.61), confirming its suitability for use (Hernández-Moreno and Landero-Hernández 2014). In our research, we obtained Cronbach’s alpha value of 0.7 and a McDonald’s ω of 0.71, indicating good internal consistency (Kaplan and Saccuzo 2009; Murphy and Davidshofer 2005; Celina Oviedo and Campo-Arias 2005).

The Oxford Happiness Inventory (OHI) was created by Hills and Argyle (2002). It was used to preserve reliability as an equivalent measure to the previous one (SHS). It measures personal happiness as the frequency of joy, average general life satisfaction, and absence of negative feelings. It consists of 29 Likert-type items with 6 response alternatives each (ranging from 1—strongly disagree to 6—strongly agree). Its original version has high internal consistency (Cronbach’s alpha of 0.90) and a test–retest correlation at seven weeks of 0.78, justifying its use in the Spanish population (Aradilla-Herrero et al. 2014). In our research, we obtained a Cronbach’s alpha value of 0.79 and a McDonald’s ω of 0.81, indicating good internal consistency and a moderate test–retest correlation (r = 0.68) (Kaplan and Saccuzo 2009; Murphy and Davidshofer 2005; Celina Oviedo and Campo-Arias 2005).

The Satisfaction with Life Scale (SWLS) was created by Diener et al. (1985), with the Spanish version for university students created by Garrido Muñoz de Arenillas et al. (2010). It is a self-report test that conceptually complements the happiness scale and evaluates cognitive aspects of SWB—specifically, the degree of satisfaction that the individual perceives from their life as a whole. It assumes that this judgment depends on comparisons the subject makes between their life circumstances and the standard they consider appropriate. It consists of 5 Likert-type items on a 7-point scale, where 1 is “completely disagree”, and 7 is “completely agree”. It has satisfactory psychometric properties. The internal consistency of the original scale was adequate (α = 0.84) and similar to that obtained in other studies such as those by Atienza et al. (2000) (α = 0.84); Cabañero Martínez et al. (2004) (α = 0.82); and Reig et al. (2001) (α = 0.83). In our research, it was good (α = 0.81, ω = 0.82) (Kaplan and Saccuzo 2009; Murphy and Davidshofer 2005; Celina Oviedo and Campo-Arias 2005), and in line with the reference studies.

Overall Life Satisfaction (OLS) was created by Campbell et al. (1976). It was used to preserve reliability as an equivalent measure to the SWLS. It is a single-item Likert scale from 0 to 10, which evaluates the subject’s subjective life satisfaction through the following question: To what extent are you satisfied with your life in general? It has been used in positive psychology research, cross-cultural studies (Anthimou et al. 2021; Berrios-Riquelme et al. 2021; De Almeida Cardoso et al. 2023), evaluations of social programs, public policies, and health research (Diener 2000; Kapteyn et al. 2014; Pavot and Diener 1993) and education (Fernández-Berrocal et al. 2022). It has high internal consistency (α > 0.80, ω = 0.88) and good construct and convergent validity with other SWB measures such as the SWLS (Diener et al. 1985, 2018; Pavot and Diener 1993).

All these instruments are widely used (Muñoz Umaña 2007), suitable for the model, easy to administer, and have good psychometric properties (validity and consistency), proven with university samples and in different cultural contexts, including Spanish (Berrios-Riquelme et al. 2021; Sánchez-Ruiz 2020; Suyo-Vega et al. 2023). Two measures were used for each SWB variable (happiness and life satisfaction) as test–retest measures to check the stability of the subjective measure.

Finally, AA was assessed in terms of subject-specific competencies and learning outcomes and as an end-of-course grade point average (GPA), one of the most commonly used measures in university educational research due to its ability to provide a quantitative and comparative evaluation of academic performance over time (Feldon et al. 2024; Madigan 2019; Richardson et al. 2012).

### 2.3. Data Collection Procedure

Informed of the study’s objectives, the students voluntarily agreed to participate and received appropriate instructions for each test. Alongside informed consent, the anonymity and confidentiality of the responses were guaranteed (APA 2020; World Medical Association 2023), as well as adherence to the ethical principles for human research outlined in the Helsinki Declaration (CIOMS 2021) and those approved by the Research Committee of our institution.

Pre-test/post-test assessments were conducted using the same instruments, simultaneously in both groups, before and after the intervention with the experimental group, and in three sessions of approximately 30 min each (EI, SWB, and AA).

The intervention, designed ad hoc, aimed to develop students’ EI and SWB (Sánchez-Ruiz 2020). It was conducted over 4 months (February-June), with a similar dose for all students, in the usual classroom, during school hours, and integrated within educational and health-related subjects. It consisted of 17 positive emotional education activities. It was conducted in 8 groups of 18–23 students each, over an average of 6 sessions, each lasting 60 min, using group dynamics such as brainstorming, cooperative learning, role-playing, pair work, image association, information search, critical reflection, debate, case studies, emotional fans, emotional self-awareness, communication, emotional repair, empathy, etc. The sessions were structured as follows: statement of objectives and development and evaluation of the activity in terms of experience and learning. The program implementation followed these steps: (a) prior training (first semester of the course) of the 6 participating teachers in positive emotional management techniques; (b) cooperative work sessions for designing tasks and techniques to be developed in the classroom; (c) consensus sessions, selection, and adaptation of activities to the group and the subject content within the curriculum; (d) follow-up sessions; and (e) final evaluation sessions.

The control group had pre-test/post-test measures at similar times as the experimental group and conducted their classes with a traditional methodology, based on a disciplinary approach, conceptual learning, information and responses, and more focused on the teacher and verbal transmission of content than on the student and active learning construction.

### 2.4. Data Analysis

To understand the sociodemographic characteristics of the sample and the levels of EI, happiness, life satisfaction, and AA, we used descriptive statistics (frequencies, percentages, means, and standard deviations). To improve the internal validity of the study, reduce sample selection bias, and increase the likelihood that the groups were comparable at the start of the experiment, following the recommendations of Campbell and Stanley (1966) and Stuart and Rubin (2008), the assignment of participants to the experimental/control condition was random and prior to data collection, respecting the intact classroom system, and controlling for the placebo and Hawthorne effects (McCarney et al. 2007) with a double-blind masking system (both the group assignment and the statistical treatment of the data were conducted independently of this information) (Molina Arias and Ochoa Sangrador 2014). As additional control measures, we preserved group equivalence through homogeneity tests (Campbell and Stanley 1966). We also ensured external validity by conducting measurements in situations similar to those in which the results were to be generalized (ecological validity) and improved the reliability of our data with measures of environmental consistency (intact classrooms and control of external variables such as the equality of the teaching or learning environment) and measurement consistency (with internal consistency tests of our evaluation instruments with more than one item, test–retest repetitions to verify the stability of the results over time, and reliability measures such as equivalence—parallel forms—as well as predictive validity—pre-test/post-test evaluations and regression analysis—and concurrent validity, using two measurement instruments for the less stable variables over time, such as happiness and life satisfaction). Normality was assumed by the central limit theorem (Martínez-González et al. 2020), homoscedasticity was calculated with Levene’s test, and the effectiveness of the intervention in the pre-test/post-test measurements (within-subjects factor) by group (between-subjects factor) was ensured with contrast statistics (repeated measures ANOVA) and their corresponding sphericity tests (Mauchly), Huynh-Feldt corrections for adjusting degrees of freedom, and precision of multivariate tests (Wilks’ Lambda), calculations of the main within/between-subjects effects and pairwise comparisons (post hoc) with Bonferroni adjustment, to identify which groups/times (pre/post-test) differed from each other. For interpretation, we considered the descriptive statistics, the effect size for each main effect and interaction (partial eta squared, *ηp*^2^), and the observed statistical power (>0.80), to avoid type II errors and ensure the power to detect significant effects. To study the differences in AA by time and group, we lacked the pre-test measurement, so we could only analyze the differences by group using Student’s *t*-tests for independent samples and their effect size (Cohen’s *d*).

The relationship between EI, happiness, life satisfaction, and AA was studied using bivariate correlations (*Pearson*), which quantified the degree of linear relationship between them, as well as their direction (positive or negative) and intensity.

To identify predictors of AA, we used a multiple linear regression analysis to forecast AA (DV) based on our students’ results in EI, happiness, and life satisfaction (IV). This analysis was first performed using the forced entry method (Enter) and then stepwise method, introducing possible predictors in order of importance. Before interpreting the coefficients, the assumptions of goodness of fit and the predictive model were evaluated. For the former, the *F*-test was used, indicating that the linear relationship was statistically significant. For the latter, the assumptions of non-collinearity, linearity, independence of errors, homoscedasticity of residuals, and normality, as well as the influence of outliers, were evaluated (Pardo and San Martín 2010). The assumption of non-collinearity was verified with the variance inflation factor (values < 10). The assumption of linearity was estimated with second- and third-order polynomial regressions, concluding that if the variables were not significant, the predictors were linearly related to the response variable. The assumption of independence of errors was calculated with the Durbin–Watson statistic (values between 1.5 and 2.5 reaffirmed this independence). The assumption of homoscedasticity was established with scatter plots of predictions and residuals, and the Breusch–Pagan test was used to check the homogeneity of residuals. In all models, the assumptions were met, except for normality. The influence of outliers was tested using Cook’s distance, which determined that there were no influential cases (values < 1) (Field 2013). To see the contribution of personal variables to AA, the adjusted *R*^2^ and the effect size of the model were calculated using Cohen’s *f*^2^ statistic, which was interpreted using the criteria (Cohen 1988): very small (<0.02), small (0.02–0.14), medium (0.15–0.34), large (>0.35). The statistical power of the regression analysis was estimated with values above the optimal 80%. To determine the individual contribution of each predictor, the average *R*^2^ statistic was used (Groemping 2006).

All statistical analyses were performed using the IBM-SPSS statistical software (v. 29 for Windows), assuming most of the time a precision margin of 95% and an error level of 5% (α = 0.05) and, in some cases, 1% (α = 0.01) and 99%, respectively.

## 3. Results

### 3.1. Initial Levels of Emotional Intelligence, Subjective Well-Being, and Academic Achievement

In Table 1, we present the baseline levels of EI, SWB, and AA. As can be seen, the initial average total EI of our students (*M* = 27.4), as well as its dimensions, were medium–high on a scale of 0–40, highlighting, in this order, emotional repair, which has the highest means (*M* = 28.09), followed by clarity (*M* = 27.19) and emotional attention (*M* = 26.94). This indicates that the evaluated students can manage and regulate their emotions; identify and understand them; and pay attention to them, which can have a positive impact on their emotional and mental well-being.

The average happiness evaluated with the OHI Inventory was 4.51 points (on a scale of 1–6) and 5 points (on a scale of 1–7) with the SHS Scale, which can be interpreted as the perception of happiness of the students evaluated at the initial moments of the study being medium–high.

Similarly, the average Life Satisfaction evaluated with OLS Scale was 7.61 points (on a scale of 1–10), and 5.37 points (on a scale of 1–7) with SWLS, which can be interpreted as medium–high scores in both cases.

Finally, it can be said that the average AA of our students was 6.73 points out of 10 (Table 2), indicating a final course grade that is medium–high, rated in Spain as “notable”.

### 3.2. Relationship Between Emotional Intelligence, Subjective Well-Being, and Academic Achievement

Upon analyzing the relationship between variables over time (pre-test/post-test), we observed the following results (Table 2).

The pre-test revealed a moderate positive relationship between the two happiness measurements and the two life satisfaction measurements, between clarity and emotional repair and happiness, between emotional clarity and life satisfaction, and between happiness and life satisfaction; it revealed a very weak positive relationship between AA/emotional clarity, and between AA/life satisfaction.

In the post-test, the previous relationships are maintained and, unlike the pre-test, a new positive, although very weak, relationship is found between AA and emotional attention and between AA and happiness (measured with Oxford). Lastly, the positive relationship (from low to moderate) between emotional repair and life satisfaction improves.

All this indicates that those who adequately understand and identify their emotions also regulate them well. Happier students are also more satisfied with life. Students with higher EI scores, particularly in clarity and emotional repair, and to a lesser extent in emotional attention, also tend to report greater happiness and life satisfaction. And, although the relationship is not very strong, students with higher AA scores also score higher in emotional attention and clarity, and are able to better understand their emotions, feel and express their feelings appropriately, while feeling happier and more satisfied with life. Considering these changes, from pre-test to post-test, we will need to contrast the extent the intervention had any effect on these results.

### 3.3. Differences by Group and Time in Emotional Intelligence, Subjective Well-Being, and Academic Achievement

#### 3.3.1. Emotional Intelligence

When evaluating the differences in EI by group and time (pre/post-test), the repeated measures ANOVA conducted (Table 3) showed significant differences in total EI and all its dimensions (emotional attention, clarity, and repair) (*p* < 0.001) with effect sizes close to medium in attention (*η*^2^ = 0.056, *β* − 1 = 0.98), clarity (*η*^2^ = 0.068, *β* − 1 = 0.99), and repair (*η*^2^ = 0.050, *β* − 1 = 0.97), and close to large in total EI (*η*^2^ = 0.089, *β* − 1 = 1), with higher mean scores in the post-test compared to the pre-test.

Regarding the group, we observed higher mean scores in the experimental group compared to the control group. However, in this case, the differences by group were only significant in clarity (*F*_(1,298)_ = 12.20, *p* < .001) and total EI (*F*_(1,298)_ = 9.06, *p* = 0.003), with small effect sizes (clarity *η*^2^ = 0.039, *β* − 1 = 0.936; total EI *η*^2^ = 0.03, *β* − 1 = 0.851). Students had different mean scores in clarity and total EI by group, with higher scores in the experimental group compared to the control group.

In the Time*Group interaction, we found statistical significance in attention (*F*_(1,298)_ = 5.19, *p* = 0.023, *η*^2^ = 0.017, *β* − 1 = 0.622), regulation (*F*_(1,298)_ = 3.90, *p* = 0.049, *η*^2^ = 0.013, *β* − 1 = 0.504), and total EI (*F*_(1,298)_ = 4.31, *p* = 0.039, *η*^2^ = 0.014, *β* − 1 = 0.543), with small effect sizes. These aspects of EI differ between pre-test/post-test times and between groups. Students in the experimental group had higher mean scores in attention, regulation, and total EI than those in the control group, and higher mean scores in the post-test than in the pre-test, indicating a significant improvement in EI after the intervention.

#### 3.3.2. Happiness

When evaluating the differences in happiness by group and time (pre/post-test), the repeated measures ANOVA conducted (Table 4) showed significant differences in the two tests used (OHI and SHS) (*p* < 0.001) with small effect sizes (OHI *η*^2^ = 0.041, *β* − 1 = 0.94; SHS *η*^2^ = 0.049, *β* − 1 = 0.97). The mean happiness scores in both tests were different by time and higher in the post-test than in the pre-test.

Regarding the group, we observed higher mean happiness scores in the experimental group compared to the control group. However, in this case, the differences by group were only significant when evaluated with the OHI test (*F*_(1,298)_ = 5.23, *p* = 0.022, *η*^2^ = 0.017, *β* − 1 = 0.631) and had a small effect size.

In the Time*Group interaction, we found statistical significance only in happiness measured with the OHI (*F*_(1,298)_ = 6.29, *p* = 0.013, *η*^2^ = 0.021, *β* − 1 = 0.705) with a small effect size. In this evaluation, happiness differs between pre-test/post-test times and between groups. Students in the experimental group had higher mean happiness scores than those in the control group, and higher in the post-test than in the pre-test, indicating a significant improvement in happiness measured with this test after the intervention.

#### 3.3.3. Life Satisfaction

When we evaluated the differences in life satisfaction by group and time (pre/post-test), the repeated measures ANOVA conducted (Table 5) showed significant differences by time in the two tests used (OLS *F*_(1,298)_ = 4.67, *p* = 0.031, *η*^2^ = 0.015, *β* − 1 = 0.577; SWLS *F*_(1,298)_ = 11.64, *p* < .001, *η*^2^ = 0.038, *β* − 1 = 0.925), with small effect sizes. The mean life satisfaction scores in both tests were different by time and higher in the post-test than in the pre-test.

Regarding the group and the Time*Group interaction, although we observed higher mean life satisfaction scores in the experimental group compared to the control group, and in the post-test compared to the pre-test, the differences were not significant. The life satisfaction of the students increased after the intervention in both groups, but the improvement was not significant.

#### 3.3.4. Academic Achievement

The differences in AA could only be studied by group and not by time (pre/post-test) because we lacked pre-test measurement. The data from the independent samples t-test calculated and their corresponding effect sizes can be seen in Table 6.

As can be observed, although the experimental group had a slightly higher mean AA (*M* = 6.85) compared to the control group (*M* = 6.58), the differences were not significant.

### 3.4. Emotional Intelligence, Happiness, and Life Satisfaction as Predictors of Academic Achievement

Our correlation data showed in the pre-test a positive and low relationship between AA and emotional clarity and AA and life satisfaction (OLS), and no relationship between AA and happiness (Table 2). In the post-test, although slightly higher, the positive and low relationship between AA and emotional clarity and between AA and life satisfaction (OLS and SWLS) remained, and a new positive low relationship appeared between AA and emotional attention, AA and happiness (OHI), and AA and life satisfaction (SWLS), which was not present in the pre-test (Table 3).

Considering the statistical significance of these correlations, we studied the predictive nature that clarity and emotional attention, life satisfaction (OLS and SLWS), and happiness (Oxford) (IV) might have on AA (DV), through a multiple linear regression analysis. We first used the forced entry method (enter) and then the stepwise method, introducing the variables in order of importance, to obtain a more comprehensive, precise, and useful understanding for more complex environments. In Table 7 and Table 8, we can see the models obtained with a significance level of α < 0.05.

The coefficient of determination R^2^ indicates that the shared variance of the variables emotional attention, clarity, life satisfaction (OLS and SWLS), and happiness (OHI), can predict/explain 2.5% of AA, with a small effect size (f^2^ = 0.03) (Table 7). The model meets the assumption of independence of errors (Durbin–Watson = 1.679), and the resulting ANOVA significantly improves the prediction of our dependent variable (AA) (*F* = 2.511; *p* = 0.03). Therefore, all the considered variables contribute to its prediction.

If we analyze the Beta coefficients, the variable with the greatest explanatory power for AA is, proportionally, emotional attention (*B* = 0.142), followed by life satisfaction (OLS *B* = 0.091; SWLS *B* = 0.071), and inversely, emotional clarity (*B* = −0.024) and happiness (*B* = −0.009). The collinearity statistics for this joint covariance show that the model meets the assumption of no collinearity. However, if we look at the statistical significance of the individual coefficients of these variables in the model (*t*-test), we see that emotional attention, with a small effect size (*f*^2^ = 0.02), is the only one with statistical significance and alone explains 2% of AA.

When we run the regression model only for the variables that are statistically significant using the stepwise method, we confirm that, of all the variables evaluated in the previous model, only emotional attention and life satisfaction (OLS) were statistically significant (Table 8).

The coefficient of determination (*R*^2^) indicates that in model 1, emotional attention predicts/explains 2% of AA with a small effect size (*f*^2^ = 0.02), and jointly (model 2) with life satisfaction (OLS), with a small effect size (*f*^2^ = 0.030), 3.2% (2% from attention and 1.2% from life satisfaction) of AA. The final model meets the assumption of independence of errors (Durbin–Watson = 1.687), and the resulting ANOVA confirms that both models significantly improve the prediction of our dependent variable (AA) (*F*_a_ = 7.242; *p* = 0.008; *F*_b_ = 5.987; *p* = 0.003).

If we look at the Beta coefficients of model 1, we see that emotional attention alone has explanatory/predictive power for AA (*B* = 0.034), and in model 2, the variable with the greatest explanatory power for AA is life satisfaction (*B* = 0.130), followed by emotional attention (*B* = 0.029). The collinearity statistics show that the model meets the assumption of no collinearity, and the independent samples *t*-test shows that there is statistical significance in the mean differences in both variables. The Pearson correlation revealed a positive dependency relationship between them, leading us to interpret that greater emotional attention and happiness are associated with higher AA. Model 1 predicts that when emotional attention increases by one unit, AA will increase by 0.034 points, while model 2 indicates that both emotional attention and life satisfaction have direct impacts on AA, with life satisfaction having a stronger impact, so that when life satisfaction increases by one unit, AA will increase by 0.130 points, and when emotional attention increases, it will increase by 0.029 points. The statistical significance of the individual coefficients of these variables in the model (*t*-test) confirms this for both variables.

## 4. Discussion and Conclusions

The objective of our research was to understand the EI, SWB, and AA of our university students; to analyze if these variables were related to each other, especially after the intervention; to find possible differences in these variables by group, due to the impact of a positive emotional intervention in the classroom compared to the use of a traditional methodology; and to verify if the evaluated EI and SWB were predictive variables of our students’ AA, in order to promote sustainable and inclusive changes at the university that create creative, positive, and healthy learning environments, and foster these competencies as facilitators of performance and employability.

Our findings support our first hypothesis, indicating that students demonstrate moderate-to-high levels of EI and are capable of effectively identifying, understanding, and regulating their emotions, in line with findings from other researchers (Rábago de Ávila et al. 2019; Gonzaga et al. 2024; Llanos Bardales et al. 2023). Their strength lies in their ability to effectively regulate and identify and express emotions, but they need to improve their emotional attention, as it scores lower compared to repair and emotional clarity. This profile suggests that the university students in our sample are competent in managing and understanding their emotions once they recognize them but could benefit from developing greater emotional awareness to identify and address their emotions more quickly.

Their scores in SWB, in its components of happiness and life satisfaction, are also medium–high, in line with other research (Abdullah et al. 2022; Liang and Sun 2022; Rábago de Ávila et al. 2019; Ross et al. 2019; Savari et al. 2023), as is their AA, which is medium–high, consistent with other studies (Barrera Hernández et al. 2018; Niño-González et al. 2017; Sánchez-Ruiz 2020) that have reached the same results.

These medium–high results in EI, SWB, and AA are indicators of a very promising personal and professional profile. EI facilitates the understanding and management of one’s own and others’ emotions, thus improving interpersonal relationships, stress management, and decision-making (Hollerer and Kohl 2022). SWB, in turn, is closely related to better mental health (Alonso-Aldana et al. 2020; Diener et al. 2018), higher productivity, and a superior quality of life, as people with high levels of well-being tend to be happier and more satisfied with their lives (Çakir and Demirel 2019; Navarro et al. 2021). Regarding performance, good performance not only implies greater efficiency and effectiveness in task completion but also opens doors to new opportunities and contributes to greater personal satisfaction and self-esteem (Fernández-Berrocal et al. 2022; García Morales 2022; Navarro et al. 2021; Obispo-Salazar et al. 2022). As can be seen, EI, SWB, and AA are interconnected (Puertas-Molero et al. 2020), and their improvement, even beyond these levels, can lead to a more balanced, productive, and satisfying life. Promoting them facilitates academic and professional success, as well as overall well-being (Brackett et al. 2011; MacCann et al. 2020).

Regarding the relationship between variables, we were able to confirm, as we proposed in our second hypothesis and as has been established in other research, that the relationships are positive and moderate. Those who understand and identify their emotions well also regulate them well (Extremera-Pacheco et al. 2019). The happiest students are the most satisfied with their lives (Çakir and Demirel 2019; Sánchez-Álvarez et al. 2020a, 2020b). Students with high EI scores also score high in happiness and life satisfaction (Salovey et al. 2002), and the relationship between AA and EI and SWB is positive (Sánchez-Álvarez et al. 2020a, 2020b; Ye et al. 2024) and ranges from low (Akdeniz and Yaizi 2023) to moderate (Llamas-Díaz et al. 2023; Sánchez-Álvarez et al. 2020a, 2020b; Trang et al. 2023). Students with higher AA scores also score higher in emotional attention and clarity, are better able to understand their emotions, and feel and express their feelings appropriately, while also feeling happier (Alves et al. 2019; Niño-González et al. 2017) and more satisfied with life (Akpur 2020; Barrera Hernández et al. 2018; García Morales 2022).

These results increase their chances of personal and social development, quality of life, health, and well-being, as well as their ability to succeed in various areas of life, including academic and professional domains (Corredor et al. 2020; Diener et al. 2018; Fernández-Berrocal et al. 2004; García Morales 2022; Navarro et al. 2021; Obispo-Salazar et al. 2022; Sánchez-Álvarez et al. 2020a, 2020b; Ye et al. 2024). Hence, we advocate for the need to incorporate these competencies into university curricula and for more research to provide data for effective interventions based on empirical evidence, considering, as Cobo-Rendón et al. (2020) stated, that there is still much to develop.

Hypothesis three, regarding differences by time and group, in favor of the post-test and the experimental group, due to the developed intervention program, was confirmed for EI (total and the dimensions of emotional attention and repair) and happiness (OHI) but not for life satisfaction or AA. In both cases, the effect size was small but had sufficient significance and statistical power, indicating the impact of the intervention on these variables. The data on emotional clarity and attention and its effect size align with those found by Brackett et al. (2011), Durlak et al. (2011), Fernández-Berrocal et al. (2022), Gorgens-Ekermans et al. (2015), Jdaitawi et al. (2011), MacCann et al. (2020), Puertas-Molero et al. (2020), and Taylor et al. (2022). The data on happiness and its effect sizes align with those found by Navarro et al. (2021), Suárez and Marrero (2020), and Puertas-Molero et al. (2020). The data on no significant differences in life satisfaction differ from those obtained by Brackett et al. (2011), Durlak et al. (2011), Fernández-Berrocal et al. (2022), Puertas-Molero et al. (2020), Suárez and Marrero (2020), Ye et al. (2024), and Taylor et al. (2022). The data on no differences in AA coincide with those found by Junça Silva and Almeida (2023), Puertas-Molero et al. (2020), Sánchez-Ruiz (2020), and Ye et al. (2024).

The meta-analysis by Quílez-Robres et al. (2023), MacCann et al. (2020), and Sánchez-Álvarez et al. (2020a, 2020b) find, in line with our results, a tendency for these variables to improve following interventions for their development, and small combined effect sizes for AA. This demonstrates the limited but significant impact of intervention programs like ours on these variables, which are cost-effective, as noted by Durlak (2016), due to their personal, academic, and professional benefits.

Finally, hypothesis four, which aimed to demonstrate that EI, happiness, and life satisfaction were predictive variables of AA, was confirmed for the dimensions of emotional attention and clarity, followed by life satisfaction and happiness (OHI). Together, although with a small effect size, they explained 2.5% of the AA of our students. Of all these variables, the one with the greatest statistical significance was emotional attention, which individually explained 2% of AA. Model 2 of the regression analysis added to the previous model the joint statistical significance of life satisfaction with emotional attention, which explained/predicted 3.2% of AA (2% from attention and 1.2% from life satisfaction). Studies such as those by MacCann et al. (2020) confirm the 2.3% variability of AA motivated by EI, particularly by emotional attention and repair, which explained an additional 3.9% and 3.6%, respectively; those by Llanos Bardales and Machuca Cabrera (2023), 2%; those by Akin and Akin (2016) and Salehi et al. (2013), 1.8%; and those by Navarro et al. (2021), with the 1.2% derived from happiness.

These findings reinforce, in the evaluated university academic context, the relevance of EI (attention) and SWB (particularly in its cognitive component of life satisfaction) as predictive variables of between 1.2% and 3.9% of AA, which can have important implications for the development of educational interventions in the field of higher education (Llanos Bardales and Machuca Cabrera 2023).

Although the predictive nature of EI and SWB on AA is small (2.5% in model 1, and 3.2% in model 2) in our research, these findings align with previous research in the field. The relevance of these variables for personal development and academic and professional success should not be underestimated. The complexity of these interactions and the moderate effects observed indicate that there is much more to discover and understand (Cizelj 2024), leaving the door open for new replications of our results.

Research has shown that emotionally intelligent people also enjoy multiple benefits in their personal lives, not just academically and professionally. They have better physical and mental health, are happier, are more satisfied, and use more adaptive emotional regulation strategies (Diener et al. 2018; Fernández-Berrocal et al. 2022; García Morales 2022).

Our study has empirically demonstrated that EI, SWB, and AA are interconnected, as Puertas-Molero et al. (2020) stated, and that this competency-based education focused on EI and SWB is both possible and necessary, having a small but significant impact on AA. Considering that these variables act as determining/explanatory factors of academic success and potentially future professional development (Diener et al. 2018; Ragusa et al. 2022; Velásquez et al. 2024), it would be essential for educational institutions in general, and universities in particular, to take measures to develop these competencies in their curricula (Alonso-Aldana et al. 2020). If universities aspire to produce graduates not only with knowledge but who are also adaptable, resilient, emotionally competent, and competitive in the job market, the integration of socio-emotional skills, such as EI and SWB, should no longer be optional but essential.

However, our work is not without limitations. These are related to the difficulties in conceptualizing our variables, as they are broad, complex, and multifaceted terms; the diversity of explanatory theoretical models; and the methodological and measurement problems that this entails. This complicates the coherent integration of the variables into a single theoretical framework (Salas-Blas 2019) and the interpretation and application of the research results (Avello Martínez et al. 2019), hence our effort towards conceptual clarification, thorough literature review, and methodological rigor in experimental design, measurement selection, and statistical data treatment.

Future research could expand the sample size and improve its representativeness with more precise experimental designs. Additionally, incorporating longitudinal, causal, and multiple measurement designs, not just cross-sectional or correlational, would allow for observing stability or changes in responses over time and provide a more robust understanding of the impact of EI and SWB on AA (Hidalgo-Fuentes et al. 2024). Cross-cultural designs could also be included to enhance applicability across a wider variety of contexts (Cobo-Rendón et al. 2020; Swami et al. 2009).

Self-report measures could also be complemented with experiential sampling, interviews, diaries, physiological measures of emotional response, or diagnostic images of brain activation (Muñoz Umaña 2007). Additionally, the inclusion of other sociodemographic (age, gender, educational background, employment status, among others), cognitive, and personality variables (self-esteem, self-efficacy, achievement orientation, adaptability, critical thinking, open-mindedness, resilience, character strengths, etc.) (Alonso-Aldana et al. 2020; Caballero-García and Sánchez-Ruiz 2024; Cobo-Rendón et al. 2020; García Morales 2022) could be explored to understand their influence on competency development and their role in determining AA.

Diener et al. (2018) propose the inclusion and study of other predictors of SWB such as temperament, income, and social relationships or contextual variables such as adaptability to the university environment, academic identity, and sense of belonging. Transformational and transactional leadership styles also seem to be positively influenced by EI (Osman 2020; Zhang et al. 2020) and may be interesting to study. Intrinsic motivation, facilitation of social interaction, extraversion, or responsibility, among others, can be moderating variables with significant influences on the EI and AA link (García Morales 2022).

Authors such as Cobo-Rendón et al. (2020) encourage the design and evaluation of intervention programs like ours and offer suggestions that can complement their effect on AA, including variables such as self-regulation of learning and adaptation to university life. They recommend the inclusion of tools that promote not only academic growth but also the holistic development of the student (Caballero-García and Sánchez-Ruiz 2021, 2024; Caballero-García et al. 2019; Sánchez-Ruiz 2020).

Intervening in these variables can prevent mental health problems such as stress and anxiety, particularly in university students facing high academic demands (Durlak et al. 2011; García Morales 2022).

In the same vein, we emphasize the need for studies that evaluate the prolonged impact of this competency-based education in higher education (Bolier et al. 2013). Lorenzo Alegría (2017), reviewing the literature on the duration of programs like ours, found short programs (10–12 h and 20–30 h on average; 10 days and 3 months) and long programs (2 years), with heterogeneous results. Our program was developed over 4 months, with an average of 6 sessions of 60 min per group, and it obtained significant positive results (jointly in emotional attention and clarity, life satisfaction, and happiness, but particularly in attention and life satisfaction), with a small effect size, in line with other interventions such as those by Sin and Lyubomirsky (2009), Vally et al. (2019), respectively.

The meta-analysis by Sin and Lyubomirsky analyzed 51 interventions in a total sample of 4266 individuals, finding effect sizes between 0.31 and 0.84, with 96% of them in the expected positive direction. The meta-analysis by Bolier et al. (2013) also evidenced improvements in the 39 interventions located, 19 of them in university students, with a small but stable effect size over several months (Baños et al. 2014; Lyubomirsky et al. 2011; Sin and Lyubomirsky 2009). The impact of our intervention was, therefore, in line with existing research. It was modest but sufficiently relevant, given that the soft skills it addresses are considered an essential aspect of the comprehensive education of citizens (European Union 2016), along with other skills such as innovation and entrepreneurship. The longitudinal and multidimensional studies that we mentioned earlier could provide a more comprehensive and accurate view of how these variables interact over time and in different contexts.

Environments that favor happiness and life satisfaction promote more complete personal and social development, creating more positive and productive work climates (Navarro et al. 2021). Similarly, universities could implement programs that promote socio-emotional, transversal, systematic, and long-term skills (Caballero-García and Sánchez-Ruiz 2018, 2021, 2024; Caballero-García et al. 2019; Sánchez-Ruiz 2020), which would favor both academic success and the personal growth of students (Cobo-Rendón et al. 2020; Seligman 2011) and reduce the gap between university and the workplace. Considering that higher education precedes the working stage, it would be of interest to examine the long-term impact of these variables at the end of studies and their incorporation into professional life. Authors like Taylor et al. (2022) support the idea of the lasting effects of these competencies and their transversality.

A significant part of these studies has been conducted with students in education and social sciences; however, these programs can have a significant impact in any discipline (Caballero-García and Sánchez-Ruiz 2018, 2021, 2024; Caballero-García et al. 2019; Obispo-Salazar et al. 2022; Sánchez-Ruiz 2020). It would be interesting to explore the implementation of these programs in STEM areas, where the academic focus has been on cognitive and technical development, to compare results (Corredor et al. 2020).

Given that EI and happiness are linked to greater life satisfaction, better social relationships, and better job performance, some cultures and educational policies are considering their incorporation for decision-making at the socio-community level (Diener et al. 2018). Their impact will need to be evaluated.

Finally, in the digital age we are in, new research could explore the integration of more advanced technologies, such as artificial intelligence, to personalize the socio-emotional training of students in real time. Authors like Suárez and Marrero (2020) are conducting interventions via email and demonstrating their effectiveness. This opens another line of work with great potential.

## Figures and Tables

**Table 1:** Descriptive statistics of emotional intelligence, well-being, and academic achievement.

	Emotional Intelligence				Subjective Well-Being				
Statistics	Emotional Attention	Emotional Clarity	Emotional Repair	Total EI	Oxford (OHI)	Happiness (SHS)	Diener (SWLS)	Life Satisfaction (OLS)	Academic Achievement (AA)
M	26.94	27.19	28.09	27.40	4.51	5.00	5.37	7.61	6.73
SD	6.397	6.521	6.142	4.57	0.621	0.975	1.04	1.50	1.59
Minimum	11	10	11	14.67	2.24	1.50	1.6	1	1.80
Maximum	40	40	40	39.33	5.76	7.00	7.0	10	10

**Table 2:** Descriptive statistics and *Pearson* correlations for the study variables (pre-test/post-test).

Variable	*M*	*SD*	Moment	Emotional Attention	Emotional Clarity	Emotional Regulation	Oxford (AHI)	Happiness (SHS)	Life Satisfaction (OLS)	Diener (SWLS)	AA
Emotional Attention	26.9	6.4	Pre-test	1							
	28.3	7.32	Post-test								
Emotional Clarity	27.2	6.52	Pre-test	0.300 **	1						
	28.7	7.24	Post-test	0.509 **							
Emotional Repair	28.1	6.14	Pre-test	0.091	0.438 **	1					
	29.4	6.79	Post-test	0.291 **	0.554 **						
Oxford (OHI)	4.51	0.62	Pre-test	0.093	0.487 **	0.457 **	1				
	4.64	0.76	Post-test	0.274 **	0.590 **	0.586 **					
Happiness (SHS)	7.62	1.5	Pre-test	0.001	0.428 **	0.463 **	0.658 **	1			
	5.22	1.04	Post-test	0.166 **	0.438 **	0.466 **	0.662 **				
Life Satisfaction (OLS)	7.62	1.5	Pre-test	0.020	0.438 **	0.365 **	0.648 **	0.682 **	1		
	7.76	1.52	Post-test	0.164 **	0.478 **	0.475 **	0.637 **	0.617 **			
Diener (SWLS)	5.37	1.04	Pre-test	0.050	0.430 **	0.305 **	0.610 **	0.566 **	0.686 **	1	
	5.54	1.08	Post-test	0.173 **	0.512 **	0.454 **	0.689 **	0.602 **	0.696 **		
Academic Achievement (AA)			Pre-test	0.106	0.113 *	0.061	0.081	0.102	0.136 *	0.107	1
	6.73	1.6	Post-test	0.154 **	0.122 *	0.054	0.122 *	0.077	0.146 *	0.140 *	

*Note*: * *p* < .05. ** *p* < .01.

**Table 3:** Repeated measures ANOVA (Moment*Group) for emotional intelligence.

Measure	Intra-Subjects Factors (Moment)	Inter-Subject Factors (Group)	*M*	*SD*	Effects		*F*_(1,298)_	*p*	Partial *η*^2^	Observed Power
Attention	Pre-test	Control	26.54	6.48	Intra-subjects (Moment)	Moment	17.76	<.001 *	0.056	0.987
		Experimental	27.27	6.33		Moment*Group	5.19	0.023 *	0.017	0.622
	Post-test	Control	27.14	6.54	Inter-subject (Group)	Intersection	5698.80	<.001 *	0.950	1.000
		Experimental	29.27	7.81		Group	3.83	0.051	0.013	0.496
Clarity	Pre-test	Control	25.98	6.32	Intra-subjects (Moment)	Moment	21.90	<.001 *	0.068	0.997
		Experimental	28.22	6.54		Moment*Group	0.61	0.436	0.002	0.122
	Post-test	Control	27.26	6.13	Inter-subject (Group)	Intersection	6091.57	<.001 *	0.953	1.000
		Experimental	30.01	7.87		Group	12.20	<.001 *	0.039	0.936
Regulation	Pre-test	Control	27.93	6.54	Intra-subjects (Moment)	Moment	15.84	<.001 *	0.050	0.978
		Experimental	28.22	5.80		Moment*Group	3.90	0.049 *	0.013	0.504
	Post-test	Control	28.54	6.59	Inter-subject (Group)	Intersection	7041.28	<.001 *	0.959	1.000
		Experimental	30.04	6.91		Group	1.73	0.190	0.006	0.259
Total EI	Pre-test	Control	26.82	4.35	Intra-subjects (Moment)	Moment	29.01	<.001 *	0.089	1.000
		Experimental	27.90	4.71		Moment*Group	4.31	0.039 *	0.014	0.543
	Post-test	Control	27.65	4.63	Inter-subject (Group)	Intersection	11,026.88	<.001 *	0.974	1.000
		Experimental	29.77	6.28		Group	9.06	0.003 *	0.03	0.851

*Note*: * *p* < .05.

**Table 4:** Repeated Measures ANOVA (Moment∗Group) for Happiness.

Measure	Intra-Subjects Factors (Moment)	Inter-Subject Factors (Group)	*M*	*SD*	Effects		*F*_(1,298)_	*p*	*ηp* ^2^	Observed Power
Happiness (OHI, Oxford)	Pre-test	Control	4.46	0.63	Intra-subjects (Moment)	Moment	12.59	<.001 *	0.041	0.943
		Experimental	4.55	0.61		Moment∗Group	6.29	0.013 *	0.021	0.705
	Post-test	Control	4.49	0.65	Inter-subject (Group)	Intersection	15,684.42	<.001 *	0.981	1.000
		Experimental	4.74	0.82		Group	5.23	0.022 *	0.017	0.631
Happiness (SHS, LyL)	Pre-test	Control	4.90	0.95	Intra-subjects (Moment)	Moment	15.26	<.001 *	0.049	0.973
		Experimental	5.09	0.99		Moment∗Group	0.26	0.607	0.001	0.081
	Post-test	Control	5.09	0.97	Inter-subject (Group)	Intersection	9984.72	<.001 *	0.971	1.000
		Experimental	5.33	1.09		Group	4.53	0.34	0.015	0.564

*Note*: * *p* < .005.

**Table 5:** Repeated measures ANOVA (Moment∗Group) for life satisfaction.

Measure	Intra-Subjects Factors (Moment)	Inter-Subject Factors (Group)	*M*	*SD*	Effects		*F*_(1,298)_	*p*	*ηp* ^2^	Observed Power
Life Satisfaction (OLS)	Pre-test	Control	7.46	1.53	Intra-subjects (Moment)	Moment	4.67	0.031 *	0.015	0.577
		Experimental	7.75	1.47		Moment∗Group	0.10	0.755	<.001	0.061
	Post-test	Control	7.63	1.47	Inter-subject (Group)	Intersection	9153.79	<.001 *	0.968	1.000
		Experimental	7.88	1.55		Group	2.87	0.091	0.01	0.393
Life Satisfaction (SWLS, Diener)	Pre-test	Control	5.31	1.07	Intra-subjects (Moment)	Moment	11.64	<.001 *	0.038	0.925
		Experimental	5.43	1.00		Moment∗Group	1.45	0.230	0.005	0.224
	Post-test	Control	5.41	0.98	Inter-subject (Group)	Intersection	9354.92	<.001 *	0.969	1.000
		Experimental	5.65	1.15		Group	2.46	0.117	0.008	0.347

*Note*: * *p* < 0.005.

**Table 6:** Independent samples *t*-tests for Academic Achievement, by group.

Academic Achievement	Statistics		Levene’s Test for Equality of Variances		*t*-Test for Equality of Means		Effect Size
	*M*	*SD*	*F*_(1,289)_	*p*	*t*	*p*	*d*
Control	6.58	1.70	0.652	0.420	−1.452	0.074	1.593
Experimental	6.85	1.49					

**Table 7:** Multiple regression model for Academic Achievement, with the joint variance of Emotional Intelligence and Subjective Well-Being. Introduction method.

Model		Unstandardized Coefficients		Standardized Coefficients	*t*	*p*	95.0% Confidence Interval for *B*		Collinearity Statistics	
		*B*	Std. Error	Beta			Lower Bound	Upper Bound	Tolerance	VIF
1	(Constant)	4.778	0.629		7.601	<.001	3.541	6.015		
	Attention	0.031	0.015	0.142	2.117	.035 *	0.002	0.060	0.727	1.375
	Life Satis. (OLS)	0.096	0.088	0.091	1.090	.277	−0.077	0.269	0.463	2.158
	Diener (SLWS)	0.104	0.132	0.071	0.787	.432	−0.156	0.365	0.406	2.464
	Happiness (OHI)	−0.020	0.185	−0.009	−0.107	.915	−0.384	0.344	0.420	2.379
	Clarity	−0.005	0.018	−0.024	−0.298	.766	−0.041	0.030	0.492	2.034
	*R*^2^ (0.025)		1.576							
	*F*_(5,294)_ = 2.511					0.030 *				
	*f*^2^ (0.030)									

*Note*: Dependent variable: academic performance. * *p* < 0.005.

**Table 8:** Multiple regression model for Academic Achievement, with the joint variance of Emotional Intelligence and Subjective Well-Being. Stepwise method.

Model		Unstandardized Coefficients		Standardized Coefficients	*t*	*p*	95.0% Confidence Interval for *B*		Collinearity Statistics	
		*B*	Std. Error	Beta			Lower Bound	Upper Bound	Tolerance	VIF
1	(Constant)	5.779	0.365		15.838	<.001	5.061	6.497		
	Attention	0.034	0.012	0.154	2.691	.008 *	0.009	0.058	1.000	1.000
	*R*^2^ (0.020)		1.580							
	*F*_(1,298)_ = 7.242					0.008 *				
	*f*^2^ (0.020)									
2	(Constant)	4.892	0.549		8.918	<.001	3.813	5.972		
	Attention	0.029	0.013	0.134	2.318	.021 *	0.004	0.054	0.973	1.028
	Life Satis.	0.130	0.061	0.124	2.155	.032 *	0.011	0.250	0.973	1.028
	*R*^2^ (0.032)		1.570							
	*F*_(1,297)_ = 4.645					0.032 *				
	*f*^2^ (0.030)									

*Note*: Dependent variable: academic performance. * *p* < .005.

## Data Availability

The data that supports the findings of this study are available from the corresponding author, [P.Á.C.-G.], upon reasonable request.
